# Prevalence and molecular characteristics of polymyxin-resistant *Enterobacterales* in a Chinese tertiary teaching hospital

**DOI:** 10.3389/fcimb.2023.1118122

**Published:** 2023-04-18

**Authors:** Chenlu Xiao, Xuming Li, Lianjiang Huang, Huiluo Cao, Lizhong Han, Yuxing Ni, Han Xia, Zhitao Yang

**Affiliations:** ^1^ Department of Laboratory Medicine, Ruijin Hospital Affiliated to Shanghai Jiao Tong University School of Medicine, Shanghai, China; ^2^ Department of Clinical Microbiology, Ruijin Hospital Affiliated to Shanghai Jiao Tong University School of Medicine, Shanghai, China; ^3^ Department of Scientific Affairs, Hugobiotech Co., Ltd., Beijing, China; ^4^ Department of Clinical Laboratory, The Second Affiliated Hospital of Xiamen Medical College, Xiamen, China; ^5^ Department of Microbiology, The University of Hongkong, Hong Kong, Hong Kong SAR, China; ^6^ Department of Infection Control, Ruijin Hospital Affiliated to Shanghai Jiao Tong University School of Medicine, Shanghai, China; ^7^ Department of Emergency, Ruijin Hospital Affiliated to Shanghai Jiao Tong University School of Medicine, Shanghai, China

**Keywords:** *Enterobacterales*, polymyxin-resistance, prevalence, phenotypes, comparative genomics

## Abstract

**Introduction:**

Polymyxin-resistant *Enterobacterales* poses a significant threat to public health globally, but its prevalence and genomic diversity within a sole hospital is less well known. In this study, the prevalence of polymyxin-resistant *Enterobacterales* in a Chinese teaching hospital was investigated with deciphering of their genetic determinants of drug resistance.

**Methods:**

Polymyxin-resistant *Enterobacterales* isolates identified by matrix-assisted laser desorption were collected in Ruijin Hospital from May to December in 2021. Both the VITEK 2 Compact and broth dilution methods were used to determine polymyxin B (PMB) susceptibility. Polymyxin-resistant isolates were further characterized by molecular typing using PCR, multi-locus sequence typing, and sequencing of the whole genome.

**Results:**

Of the 1,216 isolates collected, 32 (2.6%) across 12 wards were polymyxin-resistant (minimum inhibitory concentration (MIC) range, PMB 4–256 mg/ml, and colistin 4 ≥ 16 mg/ ml). A total of 28 (87.5%) of the polymyxin-resistant isolates had reduced susceptibility to imipenem and meropenem (MIC ≥ 16 mg/ml). Of the 32 patients, 15 patients received PMB treatment and 20 survived before discharge. The phylogenetic tree of these isolates showed they belonged to different clones and had multiple origins. The polymyxin-resistant *Klebsiella pneumoniae* isolates belonged to ST-11 (85.72%), ST-15 (10.71%), and ST-65 (3.57%), and the polymyxin-resistant *Escherichia coli* belonged to four different sequence types, namely, ST-69 (25.00%), ST-38 (25.00%), ST-648 (25.00%), and ST-1193 (25.00%). In addition, six *mgrB* specific mutations (snp_ALT c.323T>C and amino acid change p.Val8Ala) were identified in 15.6% (5/32) of the isolates. *mcr*-1, a plasmid-mediated polymyxin-resistant gene, was found in three isolates, and non-synonymous mutations including T157P, A246T, G53V, and I44L were also observed.

**Discussion:**

In our study, a low prevalence of polymyxin-resistant *Enterobacterales* was observed, but these isolates were also identified as multidrug resistant. Therefore, efficient infection control measures should be implemented to prevent the further spread of resistance to last-line polymyxin therapy.

## Introduction

The emergence of carbapenem-resistant *Enterobacterales* represents a major public health threat worldwide (Nordmann et al., 2012). Currently, periodic outbreaks or endemics of *Enterobacterales* that are not susceptible to carbapenems have now been documented in hospital settings and the wider community (Mizrahi et al., 2020), and also *Enterobacterales* have been designated by WHO as high-priority pathogens (Kopotsa et al., 2019), which limits the effectiveness of treatment of *Enterobacterales* infections. In 2021, it was estimated that 9.4%–12.5% of *Enterobacterales* isolates were carbapenem-resistant in China (http://www.chinets.com/). It is more serious that *Enterobacterales* has been included among a group of multiple drug resistance (MDR) pathogens that “ESKAPE” the actions of commonly used antibiotics (Rice, 2008).

Colistin and polymyxin B (PMB), first- generation polymyxins, were first introduced into clinical practice in the late 1950s but subsequently abandoned due to concerns about nephrotoxicity (Nang et al., 2021). Given their ever-increasing resistance to all other antibiotics, such as carbapenems and aminoglycosides, polymyxins were reintroduced into treatment regimens in the early 2000s for problematic Gram-negative “superbugs” (Velkov et al., 2019). They remain an important last-line treatment as they have excellent activity against many of these problematic pathogens (Jeannot et al., 2017). Worryingly, resistance to polymyxins of numerous bacteria, including *Enterobacterales*, has been reported in both humans and animals at an alarming rate (Gales et al., 2011; Lekunberri et al., 2017; Sherry and Howden, 2018). For example, in 2021, the China Antimicrobial Surveillance Network (http://www.chinets.com/) estimated that only 4.6% of isolates in China were resistant to colistin and PMB. In many countries, polymyxins are the only accessible or affordable therapeutic option for carbapenem-resistant organisms (Satlin et al., 2020). The emergence of bacteria resistant to polymyxins, notably *Enterobacterales* such as *Klebsiella pneumoniae* (*K. pneumoniae*) and *Escherichia coli* (*E. coli*), has been attributed to their widespread usage to treat infections (Stefaniuk and Tyski, 2019). Bacterial isolates resistant to polymyxin are of great concern worldwide because they can cause life-threatening infections, particularly those that incorporate antimicrobial resistance genes (ARGs), such as extended-spectrum β-lactamases (ESBLs) and metallo-β-lactamases (MBLs), harboring mainly *bla*_KPC_, *blα*_NDM_, and *blα*_oxA-48_ like genes which have become a major challenge to public health because of limited antibiotic choice and high case-fatality rates (Capone et al., 2013; Gao et al., 2019).

The acquisition of polymyxin resistance has been attributed to mutations of *phoP/phoQ* and *pmrA/pmrB*, which are two-component regulatory systems (TCS) (Capone et al., 2013; Jayol et al., 2015; Stefaniuk and Tyski, 2019). Constitutive upregulation of the *pmrHFIJKLM-ugd* operon is known to be triggered by highly specific mutations, which lead to the covalent attachment of 4-amino-4-deoxy-L-arabinose to the lipid A component of lipopolysaccharide that is located in the outer membrane of bacteria (Moffatt et al., 2010; Jones-Dias et al., 2016; Abdul Momin et al., 2017). The *mcr* (1–10) are polymyxin-resistant genes that have been identified in *Enterobacterales* isolates, and horizontal transfer of these genes through plasmid can alter bacterial resistance to polymyxin (Antoniadou et al., 2007; Jayol et al., 2015; Baraniak et al., 2016; Bardet et al., 2017; Borowiak et al., 2017; AbuOun et al., 2018; Carroll et al., 2019; Xu et al., 2022; Zhou et al., 2022). The aims of the present study were to determine the prevalence, molecular characteristics, and antibiotic susceptibility of polymyxin-resistant *Enterobacterales* isolated from Chinese patients in a tertiary teaching hospital.

## Methods and materials

### Bacterial isolates and antibiotic susceptibility testing

The study was based on retrospective samples collected from the Department of Clinical Microbiology of Ruijin Hospital, a 3,624-bed tertiary care teaching hospital located in east China, with approximately 130,000 hospital admissions per year. Ruijin Hospital has five intensive care unit (ICU) wards, and each ward has two sections. All *Enterobacterales* isolates were collected from May to December during 2021. The isolates were analyzed for species identification using the MALDI-TOF MS system (bioMérieux, Missouri, France) and then susceptibility tests using the AST-N335 VITEK 2 Compact system (bio Mérieux, France). The antimicrobial agents investigated were amikacin (aminoglycosides), aztreonam (monobactam), cefepime, cefoperazone/sulbactam, ceftazidime, ciprofloxacin, and levofloxacin (quinolones), colistin, doxycycline, and minocycline (tetracyclines), meropenem and imipenem (carbapenems), piperacillin/tazobactam, tigecycline (glycylcycline), ticarcillin/clavulanic acid, tobramycin, and trimethoprim/sulfamethoxazole. Minimum inhibitory concentrations (MICs) of PMB and colistin were determined using broth microdilution with *E. coli* ATCC 25922 used as the quality control strain. All results (except for polymyxins) were interpreted according to the Clinical and Laboratory Standards Institute (CLSI) guidelines. For patients with multi polymyxin-resistant isolates, only the first one was used for genome sequencing in the present study. A total of 28 K*. pneumoniae* and four *E. coli* isolates were identified to be polymyxin-resistant, and we performed whole- genome sequencing on all of them. The public genomes used in this study are summarized in **Table S1**.

### Genome sequencing, assembly, and annotation

Genomic DNA of each polymyxin-resistant isolate was extracted using the method of cetyltrimethylammonium bromide (Clarke, 2009). The quantity and quality of DNA were determined using a Qubit Fluorometer (Invitrogen, USA), and the integrity was checked using the NanoDrop spectrophotometer (Thermo Scientific, Wilmington, DE, USA). The sequencing libraries were constructed using a TruSeq DNA Sample Preparation Kit (Illumina, San Diego, USA) and a Template Prep Kit (Pacific Biosciences, Menlo Park, California, USA). Genome sequencing was carried out by Shanghai Personal Biotechnology (Shanghai, China) using an Illumina NovaSeq platform with a PE150 model. *De novo* genome assembly was conducted using A5-Miseq (v20160825) and SPAdes (v3.12.0), followed by base correction using Pilon (v1.23). The gene models were predicted by Glimmer 3.02 (http://ccb.jhu.edu/software/glimmer/index.shtml). Function annotation was completed by searching databases including NR (Non-Redundant Protein Database), KEGG (Kyoto Encyclopedia of Gene and Genomes), and COG (Cluster of Orthologous Groups of proteins) using BLAST. Subsequently, the VFDB (Virulence Factors of Pathogenic Bacteria) and CARD (the Comprehensive Antibiotic Resistance Databases) were interrogated to retrieve pathogenicity genes and antibiotic-resistant genes, respectively.

### Multilocus sequence typing

The PubMLST database (https://pubmlst.org/organisms/) for multilocus sequence typing (MLST) was utilized. Seven housekeeping genes, namely, *gapA*, *infB*, *mdh*, *pgi*, *phoE*, *rpoB*, and *tonB*, were used for *K. pneumoniae* typing, whereas *adk*, *fumC*, *gyrB*, *icd*, *mdh*, *purA*, and *recA* were adopted for *E. coli* typing.

### Variant calling and phylogenetic analyses

Taking the genome of *K. pneumoniae* (GCA_008728695.1) and *E. coli* (GCA_003018455.1) as references, Snippy (https://github.com/tseemann/snippy) was employed to identify SNPs across 378 complete genomes of *K. pneumoniae* and 404 complete genomes of *E. coli* from the GenBank database (**Supplementary Table S1**). Core SNPs were concatenated and aligned using a snippy-multi script. The SNPs were further annotated by using SnpEff (https://pcingola.github.io/SnpEff/). Subsequently, a core-SNP- based maximum likelihood tree was constructed employing IQ-TREE with a GTR+I+G model and bootstrap values of 1,000 (Minh et al., 2020), and the tree was visualized using iTOL (https://itol.embl.de/) (Letunic and Bork, 2021).

## Results

### Patient demographics and characteristics of the polymyxin-resistant Enterobacterales isolates

**Figure 1:**
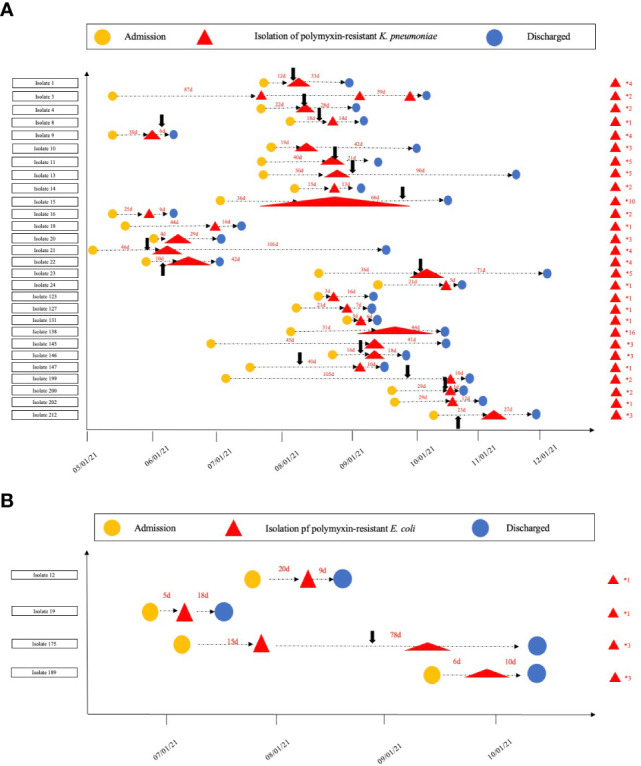
Timeline for the isolation of polymyxin-resistant (*K*) *pneumoniae* and (*E*) *coli*. **(A)**. The timeline for polymyxin-resistant (*K*) *pneumoniae* isolation in each patient; **(B)**. the timeline for the polymyxin-resistant isolation (*E*) *coli* in each patient. The numbers on the right of the red triangles show the number of polymyxin-resistant isolates from each patient. The black arrows show when polymyxin treatment commenced.

The hospitalization information, polymyxin treatment history, and amount of separated polymyxin-resistant *Enterobacterales* isolates for each patient are given in **Figure 1**. Only 28.6% (8/28) of the patients had isolates with polymyxin-resistant *K. pneumoniae* once, and the other patients had isolates multiple times. In particular, two patients were isolated with polymyxin-resistant *K. pneumoniae* isolates from one location 10 and 16 times during their hospitalization, demonstrating a persistent- infection procedure. Two patients had isolates with polymyxin-resistant *E. coli* only once; an other two patients had polymyxin-resistant *E. coli* isolates three times from one location during their hospitalization.

A total of 458 K*. pneumoniae* and 758 *E. coli* isolates were collected across the study period. Finally (28/458, 6.11%), polymyxin-resistant *K. pneumoniae* and (4/758, 0.53%) polymyxin-resistant *E. coli* strains were isolated from separate patients in 12 different wards (**Table 1**). Isolates with colistin MICs (29/32, 90.7%) > 4 μg/ml or PMB MICs that ranged from 4 to 256 μg/ml were considered to be resistant (**Table 2**). Most of the polymyxin-resistant isolates were collected from sputum (9/32, 28.1%), followed by throat swabs (4/32, 12.5%), wounds (4/32, 12.5%), blood (3/32, 9.4%), midstream urine (3/32, 9.4%), extravasate fluid (3/32, 9.4%), and bile (2/32, 6.2%). The ages of the patients ranged from 33 to 96 years (range 60.1 ± 14.3), and the majority were adults with multiple complicated comorbidities. The ward with the most frequent occurrence was the emergency intensive care unit (EICU) (9/32, 28.1%), followed by the burns ward (8/32, 25.0%), critical care unit (CCU) (4/32, 12.5%), respiratory intensive care unit (RICU) (4/32, 12.5%), gastrointestinal surgery ward (1/32, 0.1%), hematology ward (1/32, 3.1%), and urology surgery ward (1/32, 3.1%). The average length of a hospital stay was 43 days (CI: 32.25 to 64.75). Five patients (5/32,15.6%) were given tropical PMB treatment (7–22 days) and 10 patients (10/32, 31.2%) intravenous (i.v.) PMB (25–150 mg) sulfate (2–30 days). Fifteen patients (15/32, 46.9%) did not receive any polymyxin- related therapy during their hospitalization. Three patients (3/32, 9.3%) were given i.v. polymyxin E (15–30 mg/day) aerosol/sulfate therapy (4–18 days), and 2 patients (2/32, 6.2%) received PMB (50–150 mg/day) sulfate aerosol therapy (7–12 days). Most (14/15, 93.3%) polymyxin-resistance isolates originated from patients that had received polymyxin- related therapy were isolated after the polymyxin treatment (2–50 days) and only one isolate (1/15, 6.67%) of polymyxin-resistant *K. pneumoniae* was isolated before polymyxin therapy. Death due to any cause occurred in 10/32 patients, giving a mortality rate of 31.25%. Most of the polymyxin-resistant *K. pneumoniae* isolates were ST-11 (23/28, 82.14%), and the others were ST-65 (2, 7.14%) and ST-15 (3/28, 10.71%). Polymyxin-resistant *E. coli* isolates belonged to four different MLSTs, namely, ST-69, ST-38, ST-648, and ST-1193.

**Table 1:** Patient demographics and main characteristics of polymyxin-resistant *K. pneumoniae* and *E. coli*.

Isolates	Gender	Age (years)	Length of hospital stay (day)	Underlying disease	Ward	Polymyxin treatment†	Outcome	Specimen	Collection date	MLST type	Carbapenem resistance Genes
*K. pneumoniae* 1	Male	60	45	III degree alkaline corrosion injury, post-traumatic wound infection, alkaline burn of right eyeball	Burns ward	Local use of middleical polymyxin B for 7 d associated with Polymyxin B Sulfate 100 mg q12h i.v. for 7 d (same period)	Survived	Blood	2021/8/2	ST-11	*bla*_TEM-1b_, *bla*_KPC-2_, *bla*_TEM-215_, *bla*_CTX-M-65_, *bla*_Toho-2_, *bla*_SHV-2_
*K. pneumoniae* 3	Male	96	146	Sepsis due to biliary tract infection, Cholangiocarcinoma, stage IV chronic kidney disease	Respiratory	No treatment	Dead	Blood	2021/7/27	ST-15	*bla*_TEM-1b_, *bla*_SHV-28_, *bla*_CTX-M-1_, *bla*_OXA-1_
*K. pneumoniae* 4	Female	84	50	Septic shock due to intra-abdominal infection, rectal malignant tumor	EICU	Polymyxin B Sulfate 75 mg q12h i.v. for 14 d	Dead	Throat swab	2021/7/16	ST-11	*bla*_KPC-2_, *bla*_TEM-1b_, *bla*_SHV-66_
*K. pneumoniae* 8	Male	66	33	Sepsis due to systemic candidiasis and pulmonary infection (CRKP)	CCU	Polymyxin B Sulfate aerosol therapy 50 mg q12h i.v. for 8 d	Survived	Throat swab	2021/7/16	ST-11	*bla*_KPC-2_, *bla*_TEM-1b_, *bla*_SHV-66_
*K. pneumoniae* 9	Female	62	36	III degree burn, inhalation injury, post-traumatic wound infection	RICU	Local use of middleical polymyxin B for 7 d	Survived	Sputum	2021/8/23	ST-11	*bla*_KPC-2_, *bla*_TEM-1b_, *bla*_SHV-66_
*K. pneumoniae* 10	Male	71	61	Hyperosmolar coma in type 2 diabetes, with acute on chronic renal failure	Burns ward	No treatment; considered as colonization	Survived	Drainage fluid	2021/8/16	ST-11	*bla*_TEM-215_, *bla*_KPC-2_, *bla*_SHV-66_, *bla*_TEM-1b_, *bla*_CTX-M-65_
*K. pneumoniae* 11	Male	37	60	Severe acute pancreatitis, nosocomial pneumonia	EICU	Polymyxin B Sulfate 25 mg q12h i.v. for 7 d associated with Polymyxin E aerosol therapy 30 mg q12h iv for 4 d	Survived	Sputum	2021/8/16	ST-11	*bla*_TEM-215_, *bla*_KPC-2_, *bla*_SHV-66_, *bla*_TEM-1b_, *bla*_CTX-M-65_
*K. pneumoniae* 13	Male	42	120	Severe acute pancreatitis, diabetes mellitus type 2	CCU	Polymyxin B Sulfate 100 mg q12h iv for 11 d	Survived	Bile	2021/8/23	ST-11	*bla*_SHV-11_, *bla*_KPC-2_, *bla*_CTX-M-65_, *bla*_TEM-1b_
*K. pneumoniae* 14	Male	72	28	Sepsis due to urinary tract infection, acute kidney injury	EICU	No treatment; considered as colonization	Dead	Midstream-urine	2021/8/22	ST-11	*bla*_TEM-215_, *bla*_KPC-2_, *bla*_SHV-66_, *bla*_CTX-M-65_, *bla*_TEM-1b_
*K. pneumoniae* 15	Male	41	102	Severe acute pancreatitis, sepsis, complicated abdominal infection, respiratory failure, hemorrhagic shock	EICU	Polymyxin B Sulfate 50 mg q12h i.v. for 20 d	Dead	Sputum	2021/8/11	ST-11	*bla*_TEM-215_, *bla*_KPC-2_, *bla*_SHV-66_, *bla*_TEM-1_, *bla*_CTX-M-65_
*K. pneumoniae* 16	Female	62	36	III degree burn, inhalation injury, post traumatic wound infection	EICU	Local use of middleical polymyxin B for 7 d	Survived	Extravasate fluid	2021/8/11	ST-11	*bla*_TEM-215_, *bla*_KPC-2_, *bla*_SHV-66_, *bla*_TEM-1b_, *bla*_CTX-M-65_
*K. pneumoniae* 18	Female	55	60	Severe acute pancreatitis, sepsis, nosocomial pneumoni, multiple organ dysfunction syndrome	EICU	No treatment; considered as colonization	Dead	Sputum	2021/8/23	ST-11	*bla*_TEM-215_, *bla*_KPC-2_, *bla*_SHV-66_, *bla*_TEM-1b_, *bla*_CTX-M-65_
*K. pneumoniae* 20	Male	68	33	III degree burn	EICU	No treatment; considered as colonization	Survived	Extravasate fluid	2021/6/30	ST-11	*bla*_KPC-2_, *bla*_SHV-66_, *bla*_TEM-1b_, *bla*_CTX-M-65_
*K. pneumoniae* 21	Male	33	147	III degree burn, hypovolemic shock, post-traumatic wound infection	Burns ward	Local use of middleical polymyxin B for 10 d associated with Polymyxin E sulfate 30 mg q12h i.v. for 18 d	Survived	Wound	2021/6/18	ST-11	*bla*_TEM-215_, *bla*_KPC-2_, *bla*_SHV-66_, *bla*_TEM-1b_, *bla*_CTX-M-65_
*K. pneumoniae* 22	Male	43	52	III degree burn, sepsis, psoriasis	Burns ward	0.05% Local use of middleical polymyxin B for 40 d associated with Polymyxin B Sulfate 50 mg q12h i.v. for 9 d	Dead	Wound	2021/6/20	ST-11	*bla*_SHV-1a_, *bla*_TEM-215_, *bla*_TEM-1_, *bla*_CTX-M-65_
*K. pneumoniae* 23	Male	56	107	Severe respiratory failure	CCU	Polymyxin B Sulfate 150 mg q12h i.v. for 30 d	Survived	Sputum	2021/9/8	ST-11	*bla*_TEM-215_, *bla*_KPC-2_, *bla*_SHV-66_, *bla*_TEM-1b_, *bla*_CTX-M-65_
*K. pneumoniae* 24	Male	64	26	Pulmonary thromboembolism, severe pneumoniae, fungal urinary tract infection,	RICU	No treatment; considered as colonization	Survived	Sputum	2021/10/14	ST-11	*bla*_SHV-1a_, *bla*_TEM-215_, *bla*_KPC-2_, *bla*_TEM-1b_, *bla*_CTX-M-65_
*K. pneumoniae* 123	Male	61	19	Diabetic foot, peripheral neuropathy and vascular disease	Burns ward	No treatment; considered as colonization in wound	Survived	Wound	2021/6/30	ST-15	*bla*_TEM-215_, *bla*_CTX-M-15_, *bla*_TEM-1b_, *bla*_OXA-1_, *bla*_SHV-28_
*K. pneumoniae* 127	Female	59	28	Pancreatic cancer	Burns ward	No treatment; considered as colonization	Survived	Drainage fluid	2021/6/8	ST-11	*bla*_TEM-1b_, *bla*_CTX-M-15_, *bla*_TEM-215_, *bla*_SHV-66_
*K. pneumoniae* 131	Male	67	9	Stomach cancer	Gastrointestinal surgery	No treatment; considered as colonization	Survived	Sputum	2021/8/27	ST-65	*bla*_SHV-1a_
*K. pneumoniae* 138	Female	57	38	Multiple myeloma	Hematology	No treatment; considered as colonization	Survived	Throat swab	2021/8/30	ST-11	*bla*_TEM-1b_, *bla*_CTX-M-15_, *bla*_TEM-215_, *bla*_SHV-66_
*K. pneumoniae* 145	Male	33	86	Severe acute pancreatitis	EICU	No treatment; considered as colonization	Survived	Bile	2021/9/1	ST-11	*bla*_TEM-1b_, *bla*_SHV-66_
*K. pneumoniae* 146	Female	69	34	II degree burn	Burns ward	Local use of middleical polymyxin B for 22 d	Survived	Wound	2021/9/3	ST-11	*bla*_CTX-M-65_, *bla*_TEM-215_, *bla*_SHV-11_, *bla*_TEM-1b,_
*K. pneumoniae* 147	Female	84	50	Septic shock, intra-abdominal infection, rectal malignant tumor	CCU	Polymyxin B Sulfate 150 mg q12h i.v. for 14 d	Survived	Sputum	2021/9/8	ST-11	*bla*_SHV-1a_, *bla*_TEM-215_, *bla*_TEM-1_
*K. pneumoniae* 199	Male	54	76	pulmonary thromboembolism, severe pneumonia,	EICU	Polymyxin B Sulfate 50 mg q12h i.v. for 20 d	Dead	Throat swab	2021/9/9	ST-11	*bla*_CTX-M-65_, *bla*_SHV-1a_
*K. pneumoniae* 200	Female	70	30	Severe acute pancreatitis	EICU	Polymyxin B Sulfate 100 mg q12h i.v. for 2 d	Dead	Sputum	2021/10/12	ST-11	*bla*_SHV-66_, *bla*_TEM-1b_, *bla*_CTX-M-65_, *bla*_TEM-215_
*K. pneumoniae* 202	Male	59	41	Sepsis due to systemic candidiasis, myelodysplastic Syndrome	Infection Ward	No treatment	Survived	Midstream-urine	2021/11/2	ST-15	*bla*_TEM-1b_, *bla*_KPC-2_, *bla*_TEM-215_, *bla*_CTX-M-65_, *bla*_Toho-2_, *bla*_SHV-2_
*K. pneumoniae* 212	Male	72	50	Severe pneumonia, respiratory failure, urinary tract infection	RICU	Polymyxin B Sulfate 150 mg q12h i.v. for 24 d associated with Polymyxin E sulfate 15 mg q12h i.v. for 12 d	Survived	Blood	2021/11/2	ST-15	*bla*_TEM-1b_, *bla*_SHV-28_, *bla*_CTX-M-1_, *bla*_TEM-215_, *bla*_OXA-1_
*E. coli* 12	Female	71	29	Urinary tract infections	Urology	No treatment; considered as colonization	Survived	Midstream urine	2021/6/8	ST-69	*bla*_CMY-47_, *bla*_CTX-M-18_
*E. coli* 19	Female	62	36	II-degree burn, inhalation injury, post traumatic wound infection	Surgery	No treatment; considered as colonization	Survived	Drainage fluid	2021/7/11	ST-38	*bla*_CTX-M-18_, *bla*_CMY-47_
*E. coli* 175	Male	56	107	Severe acute pancreatitis, intra-abdominal infection, acute renal dysfunction	Burns ward	Polymyxin B Sulfate 150 mg q12h i.v. for 30 d	Survived	Sputum	2021/7/30	ST-648	*bla*_TEM-1b_, *bla*_OXA-10_, *bla*_CTX-M-55_
*E. coli* 189	Female	63	16	Multiple myeloma stage III	RICU	No treatment; considered as colonization	Survived	Throat swab	2021/9/29	ST-1193	*bla*_NDM-5_, *bla*_TEM-1b_

**Table 2:** Antimicrobial susceptibilities of polymyxin-resistant *K. pneumoniae* and *E. coli* isolates.

Antibiotics	Resistant isolates (whole)	Resistant *K. pneumoniae*	Resistant *E. coli*	AST range (μg/ml)
Amikacin	24 (75.00%)	21 (75.00%)	3 (75.00%)	8-64
Aztreonam	29 (90.63%)	26 (92.90%)	3 (75.00%)	2-64
Cefepime	28 (89.30%)	25 (89.30%)	3 (75.00%)	2-32
Cefoperazone/sulbactam	28 (89.30%)	25 (89.30%)	3 (75.00%)	8-64
Ceftazidime	26 (81.25%)	22 (78.60%)	4 (100.00%)	2-64
Ciprofloxacin	29 (90.63%)	25 (89.30%)	4 (100.00%)	0.5-4
Colistin	32 (100.00%)	28 (100.00%)	4 (100.00%)	≥ 4-16
Doxycycline	24 (75.00%)	21 (75.00%)	3 (75.00%)	0.5-16
Imipenem	30 (93.75%)	27 (96.40%)	3 (75.00%)	0.25-16
Levofloxacin	29 (90.63%)	25 (89.30%)	4 (100.00%)	1-64
Meropenem	28 (89.30%)	25 (89.30%)	3 (75.00%)	0.25-16
Minocycline	23 (71.88%)	21 (75.00%)	2 (50.00%)	1-16
Piperacillin/tazobactam	27 (85.70%)	24 (85.70%)	3 (75.00%)	4-128
Polymyxin B	32 (100.00%)	28 (100.00%)	4 (100.00%)	≥ 4-256
Ticarcillin/clavulanic acid	25 (78.13%)	22 (78.60%)	3 (75.00%)	8-128
Tigecycline	19 (59.38%)	16 (57.10%)	2 (50.00%)	1-8
Tobramycin	26 (81.25%)	23 (82.10%)	3 (75.00%)	1-16
Trimethoprim/sulfamethoxazole	25 (78.13%)	22 (78.60%)	3 (75.00%)	20-320

### Genetic determinants of antibiotic resistance in polymyxin-resistant isolates

All the polymyxin-resistant isolates (32/32,100%) possessed carbapenemase genes (**Table 1**). No *mcr* genes were detected in polymyxin-resistant *K. pneumoniae* isolates, showing that *mcr* genes were not the cause of polymyxin resistance in these strains. In contrast, three of four polymyxin-resistant *E. coli* isolates carried *mcr*-1 polymyxin-resistant genes (isolates 12, 175, and 189).

Specific mutations in *mgrB* were identified in 15.6% (5/32) of the isolates, including one *K. pneumonia* (isolate 123) and four *E. coli* (isolates 12, 19, 175, and 189). Taking a polymyxin-susceptible genome RJBS176 (Xu et al., 2021) as the reference, 18 mutations of *pmrA*/*pmrB* and *phoP*/*phoQ* were identified in polymyxin-resistant *K. pneumoniae* isolates, among which nine were synonymous. T157P (Bir et al., 2022) and A246T in *pmrB* and G53V in *pmrA* (Nirwan et al., 2021) were considered as variations that potentially contributed to polymyxin resistance in *K. pneumoniae* isolates (**Tables 3**, **S2**, **S3**).

**Table 3:** Genetic variations in *pmrA*, *pmrB*, *phoQ*, and *phoP* of the polymyxin-resistant *K. pneumoniae* isolates.

Isolates	*pmrB*	*pmrA*	*phoQ*	*phoP*
1	NA	NA	NA	NA
3	c.294A>T (p.Arg98Arg), c.336T>C (p.Thr112Thr), c.663G>C (p.Pro221Pro), c.736G>A (p.Ala246Thr), c.766G>C (p.Gly256Arg), c.846G>A (p.Ala282Ala), c.1032A>C (p.Pro344Pro)	NA	NA	NA
4	NA	NA	c.603_628delCAACCTGCTGCTGGTGATCCCCCTGC	c.363T> C(p.Gly121Gly)
8	NA	NA	NA	NA
9	NA	NA	NA	NA
10	NA	NA	NA	NA
11	NA	NA	c.326_349delTTCAACCGGAATGGCTGAAACGCA	NA
13	NA	NA	NA	NA
14	NA	NA	NA	NA
15	NA	NA	NA	NA
16	c.469A>C (p.Thr157Pro)	NA	NA	NA
18	c.41G>C (p.Arg14Pro)	NA	NA	c.633C>T (p.Thr211Thr)
20	NA	NA	NA	NA
21	NA	NA	NA	c.363T>C (p.Gly121Gly)
22	NA	NA	c.158G>T (p.Gly53Val)	NA
23	c.469A>C (p.Thr157Pro)	NA	NA	NA
24	NA	NA	NA	NA
123	c.294A>T (p.Arg98Arg), c.336T>C (p.Thr112Thr), c.663G>C (p.Pro221Pro), c.736G>A (p.Ala246Thr), c.766G>C (p.Gly256Arg), c.846G>A (p.Ala282Ala), c.1032A>C (p.Pro344Pro)	NA	NA	NA
127	NA	NA	NA	NA
131	c.399G>A (p.Leu133Leu), c.663G>C (p.Pro221Pro), c.736G>A (p.Ala246Thr), c.766G>C (p.Gly256Arg)	c.390C>A (p.Gly130Gly),c.615G>A (c.41G>C)	NA	NA
138	c.616G>C (p.Ala206Pro)	NA	NA	NA
145	NA	NA	NA	NA
146	NA	NA	NA	NA
147	NA	NA	NA	NA
199	NA	NA	NA	NA
200	NA	NA	c.326_349delTTCAACCGGAATGGCTGAAACGCA	NA
202	c.294A>T (p.Arg98Arg), c.336T>C (p.Thr112Thr), c.663G>C (p.Pro221Pro), c.736G>A (p.Ala246Thr), c.766G>C (p.Gly256Arg), c.846G>A (p.Ala282Ala), c.1032A>C (p.Pro344Pro)	NA	NA	c.363T>C (p.Gly121Gly)
212	c.294A>T (p.Arg98Arg), c.336T>C (p.Thr112Thr), c.663G>C (p.Pro221Pro), c.736G>A (p.Ala246Thr), c.766G>C (p.Gly256Arg), c.846G>A (p.Ala282Ala), c.1032A>C (p.Pro344Pro)	NA	NA	c.363T>C (p.Gly121Gl y)

NA, Not applicable.

When the polymyxin-resistant *E. coli* isolates’ genomes were compared with a polymyxin-susceptible reference genome (*E. coli* EC1390) (Thuy et al., 2022), we identified 137 mutations in *mgrB*, *pmrA*/*pmrB*, and *phoP*/*phoQ* including 63 synonymous ones. Among the genetic variations, I44L (Sánchez et al., 2021; Huang et al., 2021a) in *phoP* has been reported as a variation that potentially contributes to polymyxin-resistant in *E. coli* isolates. In addition, a non-synonymous mutation c.323T>C that resulted in p.Val8Ala was identified in *mgrB* of isolate 12 (**Tables 4**, **S4**, **S5**).

**Table 4:** Genetic variations in *pmrA*, *pmrB*, *phoQ*, *phoP*, and *mgrB* of the polymyxin-resistant *E. coli* isolates.

Isolate	*mgrB*	*pmrB*	*pmrA*	*phoQ*	*phop*
12	c.23T>C (p.Val8Ala)	c.1012dupT (p.Ser338fs), c.486dupG (p.Cys163fs), c.63C>T (p.Ile21Ile), c.664dupC (p.Leu222fs), c.852A>G (p.Ter284Trpext*)?, c.868A>C (p.Ser290Arg), c.886A>G (p.Arg296Gly), c.910dupG (p.Glu304fs), c.924G>A (p.Ala308Ala)	c.126A>C (p.Ala42Ala), c.210_215delTATACCinsATACACT (p.Asn70fs), c.287C>T (p.Thr96Met), c.382_383delTTinsAC (p.Leu128Thr), c.404C>T (p.Thr135Ile), c.46C>T (p.Leu16Leu), c.46C>T (p.Leu16Leu), c.560C>T (p.Pro187Leu), c.632C>A (p.Ala211Asp)	c.1035A>G (p.Ser345Serz), c.1188T>A (p.Ile396Ile), c.1203C>A (p.Thr401Thr), c.1389G>T (p.Leu463Leu), c.1415dupT (p.Leu472fs), c.1431G>A (p.Leu477Leu), c.17dupT (p.Leu8fs), c.277dupT (p.Tyr93fs), c.402T>A (p.Ile134Ile), c.470A>G (p.Gln157Arg), c.506A>G ((p.Asn169Ser), c.548dupA (p.Phe184fs), c.666dupA (p.Glu223fs), c.506A>G (p.Asn169Ser), c.548dupA (p.Phe184fs), c.60G>A (p.Ala20Ala), c.666dupA (p.Glu223fs), c.666dupA (p.Glu223fs), c.67C>T (p.Leu23Leu), c.792C>T (p.Tyr264Tyr), c.798G>A (p.Thr266Thr), c.7G>A (p.Ala3Thr), c.876A>G (p.Glu292Glu), c.900G>A (p.Glu300Glu)	c.130A>T (p.Ile44Leu), c.207C>T (p.Asn69Asn), c.279_282delCGGTinsTGGC (p.95), c.294T>C (p.Tyr98Tyr), c.386_387insAC (p.Val131fs), c.505C>G (p.Gln169Glu), c.598dupA (p.Ile200fs), c.615C>G (p.Pro205Pro), c.643delGinsCA (p.Gly215fs)
19	NA	c.1012dupT (p.Ser338fs), c.1053G>A (p.Gly351Gly), c.1064G>A (p.Arg355Lys), c.286_287delGCinsCGA (p.Glu103fs), c.354C>T (p.Glu125Asp), c.375A>T (p.Glu125Asp), c.390G>A (p.Ala130Ala), c.486dupG (p.Cys163fs), c.551G>A (p.Gly184Asp), c.664dupC (p.Leu222fs), c.679C>T (p.Gln227*), c.700A>C (p.Thr234Pro), c.808C>T (p.Gln270*), c.814G>A (p.Ala272Thr), c.852A>G (p.Ter284Trpext*)?, c.868A>C (p.Ser290Arg), c.886A>G (p.Arg296Gly), c.910dupG (p.Glu304fs), c.933T>A (p.Arg311Arg)	c.126A>C (p.Ala42Ala), c.159G>C (p.Gly53Gly), c.168C>T (p.Asp56Asp), c.177A>G (p.Gly59Gly), c.189C>T (p.Leu63Leu), c.210_215delTATACCinsATACACT (p.Asn70fs), c.224_230delACTGATCinsGTTAATT (p.TyrTerSer75CysTerPhe), c.239T>C (p.Leu80Pro), c.248_251delGCTGinsCCTT (p.ArgTer83ProLeuext*)?, c.281T>G (p.Val94Gly), c.290C>T (p.Thr97Ile), c.323_326delACATinsGCAC (p.TyrMet108CysThr), c.326T>C (p.Met109Thr), c.392T>C (p.Ile131Thr), c.404C>T (p.Thr135Ile), c.416T>C (p.Val139Ala), c.429G>A (p.Trp143*), c.434T>A (p.Val145Glu), c.440_441delGTinsAC (p.Ser147Asn), c.46C>T (p.Leu16Leu), c.416T>C (p.Val139Ala), c.429G>A (p.Trp143*), c.434T>A (p.Val145Glu), c.440_441delGTinsAC (p.Ser147Asn), c.46C>T (p.Leu16Leu), c.476_482delACGGTTAinsGCGCCTG (p.HisGly159ArgAla), c.488C>T (p.Ser163Leu), c.500T>C (p.Val167Ala), c.530C>T (p.Thr177Ile), c.536T>C (p.Ile179Thr), c.548_551delTGAAinsCGAG (p.MetAsn183ThrSer), c.584_587delCAATinsTAAC (p.ThrIle195IleThr), c.632C>A (p.Ala211Asp), c.653G>A (p.Arg218Gln), c.92_93delCAinsGC (p.Thr31Ser)	c.1018C>T (p.Leu340Leu), c.1035A>G (p.Ser345Serz), c.1086T>C (p.Ile362Ile), c.1095G>A (p.Glu365Glu), c.1107C>T (p.Val369Val), c.1119C>T (p.Asn373Asn), c.1131G>A (p.Glu377Glu), c.1320A>G (p.Val440Val), c.132T>C (p.Thr44Thr), c.1407G>A (p.Glu469Glu), c.1415dupT (p.Leu472fs), c.17dupT (p.Leu8fs), c.243C>G (p.Thr81Thr), c.249G>A (p.Thr83Thr), c.277dupT (p.Tyr93fs), c.470A>G (p.Gln157Arg), c.48G>A (p.Leu16Leu), c.506A>G (p.Asn169Ser), c.548dupA (p.Phe184fs), c.60G>A (p.Ala20Ala), c.666dupA (p.Glu223fs), c.48G>A (p.Leu16Leu), c.506A>G (p.Asn169Ser), c.548dupA (p.Phe184fs), c.60G>A (p.Ala20Ala), c.666dupA (p.Glu223fs), c.67C>T (p.Leu23Leu), c.666dupA (p.Glu223fs), c.67C>T (p.Leu23Leu), c.696C>T (p.Arg232Arg), c.757T>C (p.Leu253Leu), c.792C>T (p.Tyr264Tyr), c.876A>G (p.Glu292Glu), c.96C>T (p.Val32Val), c.987C>T (p.Leu329Leu)	c.45C>T (p.His15His), c.130A>T (p.Ile44Leu), c.165A>C (p.Pro55Pro), c.207C>T (p.Asn69Asn), c.294T>C (p.Tyr98Tyr), c.306G>A (p.Pro102Pro), c.386_387insAC (p.Val131fs), c.409T>C (p.Ser137Pro), c.505C>G (p.Gln169Glu), c.559C>T (p.Pro187Ser), c.568T>A (p.Ter190Argext*)?, c.599_600delTTinsATA (p.Ile200fs), c.615C>G (p.Pro205Pro), c.643delGinsCA (p.Gly215fs)
175	NA	c.1012dupT (p.Ser338fs), c.286_287delGCinsCGA (p.Glu103fs), c.342C>T (p.Pro114Pro), c.393A>G (p.Leu131Leu), c.419G>A (p.Ser140Asn), c.486dupG (p.Cys163fs), c.548_551delACGGinsTCGA (p.HisGly183LeuAsp), c.572C>T (p.Ala191Val), c.63C>T (p.Ile21Ile), c.664dupC (p.Leu222fs), c.852A>G (p.Ter284Trpext*)?, c.868A>C (p.Ser290Arg), c.886A>G (p.Arg296Gly), c.910dupG (p.Glu304fs), c.947A>T (p.Tyr316Phe)	c.126A>C (p.Ala42Ala), c.210_215delTATACCinsATACACT (p.Asn70fs), c.287C>T (p.Thr96Met), c.326T>C (p.Met109Thr), c.404C>T (p.Thr135Ile), c.46C>T (p.Leu16Leu), c.416T>C (p.Val139Ala), c.46C>T (p.Leu16Leu), c.60G>A (p.Ala20Ala)	c.1035A>G (p.Ser345Serz), c.1188T>A (p.Ile396Ile), c.1203C>A (p.Thr401Thr), c.132T>C (p.Thr44Thr), c.1415dupT (p.Leu472fs), c.17dupT (p.Leu8fs), c.277dupT (p.Tyr93fs), c.470A>G (p.Gln157Arg), c.48G>A (p.Leu16Leu), c.506A>G (p.Asn169Ser), c.548dupA (p.Phe184fs), c.60G>A (p.Ala20Ala), c.666dupA (p.Glu223fs), c.48G>A (p.Leu16Leu), c.506A>G (p.Asn169Ser), c.548dupA (p.Phe184fs), c.60G>A (p.Ala20Ala), c.666dupA (p.Glu223fs), c.67C>T (p.Leu23Leu), c.666dupA (p.Glu223fs), c.67C>T (p.Leu23Leu), c.792C>T (p.Tyr264Tyr), c.876A>G (p.Glu292Glu), c.900G>A (p.Glu300Glu), c.96C>T (p.Val32Val), c.975C>T (p.Gly325Gly)	c.130A>T (p.Ile44Leu), c.156C>G (p.Leu52Leu), c.159A>T (p.Gly53Gly), c.207C>T (p.Asn69Asn), c.279_282delCGGTinsTGGC (p.95), c.294T>C (p.Tyr98Tyr), c.306G>A (p.Pro102Pro), c.386_387insAC (p.Val131fs), c.526C>A (p.Pro176Thr), c.535T>C (p.Ser179Pro), c.559C>T (p.Pro187Ser), c.568T>A (p.Ter190Argext*)?, c.599_600delTTinsATA (p.Ile200fs), c.615C>G (p.Pro205Pro), c.643delGinsCA (p.Gly215fs)
189	NA	c.1012dupT (p.Ser338fs), c.1081C>T (p.Gln361*), c.339G>A (p.Thr113Thr), c.387G>A (p.Ser129Ser), c.486dupG (p.Cys163fs), c.548_551delACGGinsTCGA (p.HisGly183LeuAsp), c.572C>T (p.Ala191Val), c.582C>T (p.Ser194Ser), c.623T>G (p.Val208Gly), c.664dupC (p.Leu222fs), c.852A>G (p.Ter284Trpext*)?, c.886A>G (p.Arg296Gly), c.910dupG (p.Glu304fs),	c.126A>C (p.Ala42Ala), c.210_215delTATACCinsATACACT (p.Asn70fs), c.287C>T (p.Thr96Met), c.326T>C (p.Met109Thr), c.404C>T (p.Thr135Ile), c.46C>T (p.Leu16Leu), c.416T>C (p.Val139Ala), c.46C>T (p.Leu16Leu), c.500T>C (p.Val167Ala), c.593C>A (p.Ala198Glu), c.632C>A (p.Ala211Asp), c.653G>A (p.Arg218Gln)	c.1035A>G (p.Ser345Serz), c.1107C>T (p.Val369Val), c.1143C>T (p.Asn381Asn), c.1155T>C (p.Asn385Asn), c.1188T>A (p.Ile396Ile), c.1320A>G (p.Val440Val), c.1387C>A (p.Leu463Met), c.1415dupT (p.Leu472fs), c.17dupT (p.Leu8fs), c.277dupT (p.Tyr93fs), c.470A>G (p.Gln157Arg), c.506A>G (p.Asn169Ser), c.548dupA (p.Phe184fs), c.666dupA (p.Glu223fs), c.506A>G (p.Asn169Ser), c.548dupA (p.Phe184fs), c.60G>A (p.Ala20Ala), c.666dupA (p.Glu223fs), c.666dupA (p.Glu223fs), c.732C>A (p.Thr244Thr), c.792C>T (p.Tyr264Tyr), c.798G>A (p.Thr266Thr), c.876A>G (p.Glu292Glu), c.996G>A (p.Glu332Glu)	c.130A>T (p.Ile44Leu), c.156C>G (p.Leu52Leu), c.165A>C (p.Pro55Pro), c.174C>T (p.Asp58Asp), c.207C>T (p.Asn69Asn), c.294T>C (p.Tyr98Tyr), c.387_388delCCinsACCA (p.Val131fs), c.409T>C (p.Ser137Pro), c.526C>A (p.Pro176Thr), c.535T>C (p.Ser179Pro), c.598dupA (p.Ile200fs), c.615C>G (p.Pro205Pro), c.643delGinsCA (p.Gly215fs)

NA, Not applicable.

### Antimicrobial susceptibility and MDR of polymyxin-resistant *Enterobacterales*

The antibiotic susceptibility test result of the *Enterobacterales* isolates is shown in **Table 2**. According to the CLSI (Satlin et al., 2020), most of the polymyxin-resistant *K. pneumoniae* also showed reduced susceptibility to carbapenems, aminoglycosides, and fluoroquinolones, with 96.4% (27/28) isolates resistant to imipenem and (25/28, 89.3%) to meropenem and 75.0% (21/28) isolates to amikacin and 89.3% (25/28) to ciprofloxacin and levofloxacin. A much lower frequency (16/28, 57.1%) of tigecycline and minocycline resistance was observed. Additionally, polymyxin-resistant *E. coli* also showed decreased susceptibility to carbapenems, aminoglycosides, and fluoroquinolones. 75.0% (3/4) of isolates were resistant to imipenem and meropenem, 75.0% (3/4) to amikacin, and all of the isolates to ciprofloxacin and levofloxacin. It is also noteworthy that 50% (2/4) of the isolates were sensitive to tigecycline and minocycline. Only one polymyxin-resistant *K. pneumoniae* (isolate 199) had PMB MICs at 256 μg/ml.

### Phylogenetic analysis

**Figure 2:**
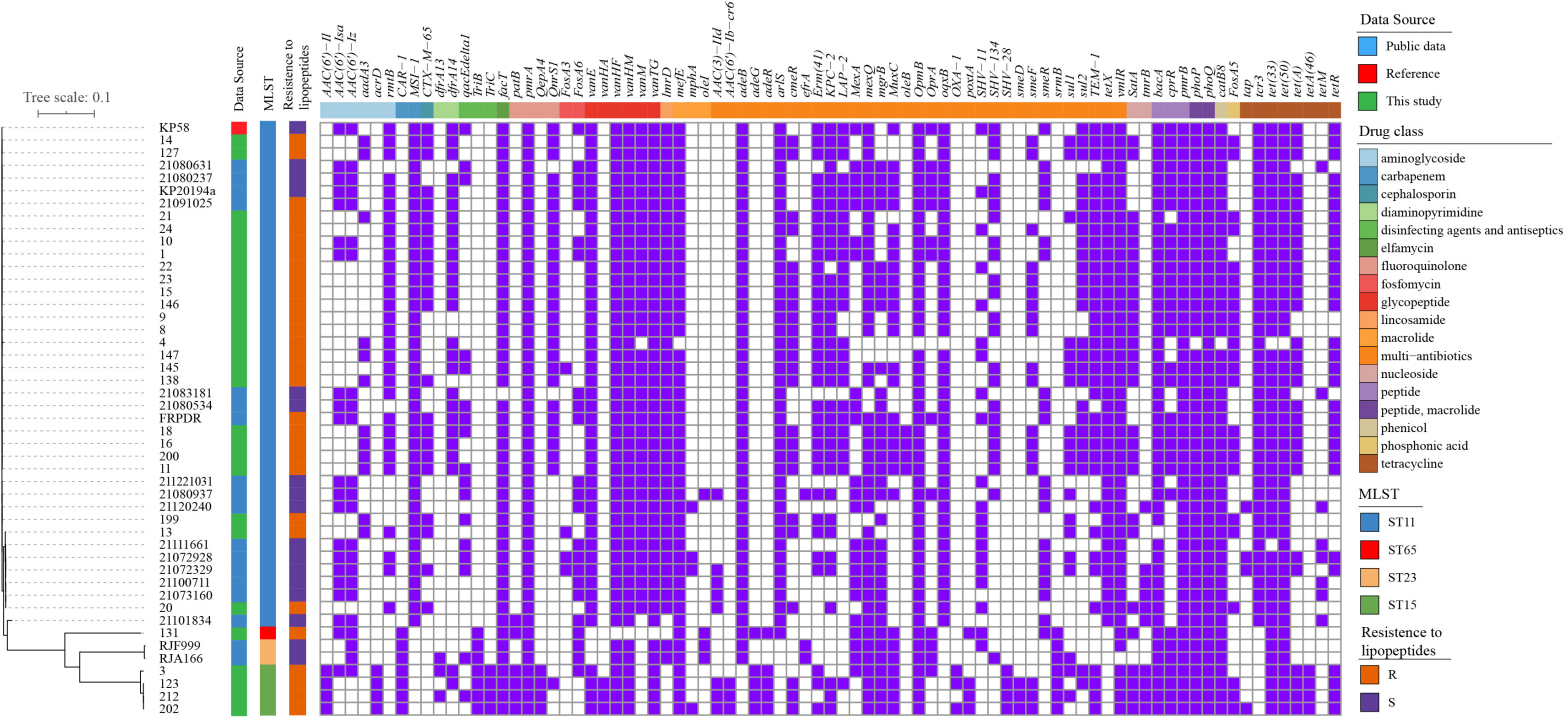
Phylogenetic tree and distribution of resistance genes of *K. pneumoniae* isolates **(A)** core-genome-based ML phylogenetic tree showing the relationship of 30 polymyxin-resistant and 17 polymyxin-sensitive *K. pneumoniae* isolates constructed taking KP58 as the reference. Apart from 28 strains sequenced in this study, the other 19 genomes were obtained from public data. The antibiotic- resistant genes were mapped on the right, with carried genes painted in purple and classified according to the ResFinder database.

Phylogenomic trees of the 28 polymyxin-resistant *K. pneumoniae* and four polymyxin-resistant *E. coli* isolates are shown in **Figure S1**, **S2**. The phylogenetic tree of *E. coli* isolates revealed that four polymyxin-resistant isolates did not belong to the same clone but had multiple origins, demonstrating their parallel evolutions. The same phenomenon was observed for *K. pneumoniae isolates*. The difference was that most of the *K. pneumoniae* isolates belonged to ST-11 with a small genetic distance. To further explore the polymyxin-resistant mechanism(s) of *K. pneumoniae*, we compared their phylogenetic relationships with polymyxin-susceptible isolates from 46 isolates and aligned antibiotic resistance genes with their phylogenetic tree (**Figure 2**). The tree shows a close relationship of isolates within the same ST.

A high- frequency appearance of *pmrA/pmrB*, *phoP/phoQ*, *cprR*, and *bacA* was observed in all isolates. Interestingly, *fosA5*, *satA*, and *cmeR* were observed in polymyxin-resistant isolates with high frequencies but were absent in all polymyxin-sensitive isolates. In reverse, *aac(6’)-Iz*, *aac(6’)-Isa*, *fosA6*, *mexA*, and *smeR* had a high frequency in polymyxin-resistant isolates but a low appearance in polymyxin-sensitive isolates. These genes may also be related to the polymyxin-resistant mechanism(s), but further molecular experiments will need to be performed for unequivocal verification.

82.14% (23/28) of the polymyxin-resistant *K. pneumoniae* isolates were detected with *mgrB* genes (**Table 5**). All the polymyxin-resistant *E. coli* isolates were detected with the *mgrB* gene, and 75% (3/4) were identified with *mcr-*1, which are located in the plasmids. Isolate 12 harbors a P0111_1 plasmid, isolate 19 harbors a IncI2_1 plasmid, and isolate 189 harbors a IncI2_1_Delta plasmid. There have been studies showing that *mcr*-1 harboring IncI plasmids were prevalent in various *Enterobacterales* in China, including animal- originated *E. coli* isolates (Tang et al., 2022; Wang et al., 2022), *Escherichia fergusonii*, and *Salmonella* (Tang et al., 2020; Tang et al., 2023), which were also observed in our study.

**Table 5:** *mgrB* and *mcr* genes in polymyxin-resistant *E. coli* isolates.

Gene	Isolates	Contig name	Scaffold affiliation	Plasmid information
*mgrB*	12	Contig1	Chromosome	–
*mgrB*	19	Contig36	Chromosome	–
*mgrB*	189	Contig2	Chromosome	–
*mgrB*	175	Contig1	Chromosome	–
*mcr*-1	12	Contig46	Plasmid	P0111-1
*mcr*-1	19	Contig37	Plasmid	IncI2-1
*mcr*-1	189	Contig29	Plasmid	IncI2-1-Delta
*mcr*-1	175	Absent	–	–

## Discussion

The world is facing the great challenge of bacterial resistance to carbapenems. This is because tigecycline and ceftazidime/avibactam have only been registered in a small number of countries and that polymyxins are often the only effective antibiotic against multidrug-resistant organisms. However, due to the widespread use of PMB and colistin against MDR organisms, such as *Acinetobacter baumannii*, *Pseudomonas aeruginosa* (*P. aeruginosa*), *K. pneumoniae*, and *E. coli*, the current low levels of polymyxin resistance are changing. Our hospital started using PMB, colistin, and corresponding susceptibility tests in late 2020 to evaluate the effectiveness of therapy that was used to treat carbapenem-resistant Gram-negative bacterial infections. There have been previous reports of infections caused by polymyxin-resistant *K. pneumoniae* (Jayol et al., 2015) and *E. coli* which carried *mcr* genes (He et al., 2017), and other studies have pooled data from multiple institutions to determine regional susceptibility to polymyxins (Wi et al., 2017; Baek et al., 2020). In the present study, we reported an investigation of the prevalence of polymyxin-resistant *Enterobacterales* during 2021 in our hospital, analyzed the isolates’ genomics and phenotypes, and compared the clinical characteristics of the patients from which they were isolated.

In our study in a single hospital, the prevalence of polymyxin resistance was 2.6% (32/1216), which is a little higher than reported (1.9%) by the China Antimicrobial Surveillance Network in 2021. Most polymyxin-resistant isolates have low MICs to PMB and colistin, which is not surprising given their structural similarity, differing only by a single amino acid (Gales et al., 2011). Of concern is that all the isolates contained OXA and β-lactamases, which confer resistance to imipenem and meropenem. Using the definitions for MDR, extensively drug-resistant (XDR), and pan drug-resistant (PDR) (Magiorakos et al., 2012), half of the polymyxin-resistant isolates were PDR and resistant to all antibiotics tested, whereas the others were XDR or MDR. ST-11, one of the first identified pandemic *K. pneumoniae* clones, has been described in Asia, Australia, USA, and Europe (Chen et al., 2014; Tian et al., 2019; Tumbarello et al., 2021; Huang et al., 2021b). Several studies have suggested that a specific ST-11 subclone with different β-lactamases might have increased its epidemic potential (Giske et al., 2012; Sonnevend et al., 2013; Tan et al., 2019). ST-11 *K. pneumoniae* was also the most dominant strain prevalent in China and exhibited reduced susceptibility to most available antibiotics (Liu et al., 2022; Ouyang et al., 2022), which is consistent with our findings. The *K. pneumoniae* ST-15 and ST-65 were reported to be prevalent mainly in Asia and also carry multiple resistance genes (Lin et al., 2014; Zhao et al., 2021). In our study, most of the prevalence isolates (ST-11) got similar plasmid types, which carry carbapenemase resistance genes. Polymyxin-resistant *E. coli* isolates in the present study were composed of multiple MLST types. ST-156 or ST-167 clones have been reported at a higher frequency in China (Xia et al., 2017), whereas ST-69 was found reported in Korea (Kim et al., 2020), ST-38 in the United Kingdom (Greig et al., 2018), ST-648 in Kerman, Iran (Kalantar-Neyestanaki et al., 2020), and ST-1193 in Canada (Izydorczyk et al., 2020). All carried multiple ARGs findings consistent with the results of the present study.

Changes in the two-component systems *pmrA*/*pmrB* (*E. coli*, *K. pneumoniae*, *Salmonella* spp.), *phoP*/*phoQ* (*K. pneumoniae*, *Salmonella* spp.), *parR*/*parS* (*P. aeruginosa*), *colR/colS* (*P. aeruginosa*), and *cprR*/*cprS* (*Campylobacter jejuni*) are associated with polymyxin resistance in Gram-negative bacteria (Baron et al., 2016). In the present study, all the polymyxin-resistant isolates had mutations in *pmrA* and/or *pmrB*, a major TCS in Gram-negative bacteria, which are responsible for polymyxin resistance. Our study indicates that the *mgrB* alteration may be a common mechanism associated with polymyxin resistance for the clinical treatment of *K. pneumoniae* infection (Cannatelli et al., 2013). However, only one of the polymyxin-resistant *K. pneumoniae* isolates contains *mgrB* resistance genes, and an intriguing aspect is that all four isolates of polymyxin-resistant *E. coli* carried *mgrB* genes and β-lactamases. Thus far, other studies have reported 10 variants of the *mcr* genes, *mcr* (1–10) in various *Enterobacterales* (Antoniadou et al., 2007; Jayol et al., 2015; Baraniak et al., 2016; Bardet et al., 2017; Borowiak et al., 2017; AbuOun et al., 2018; Carroll et al., 2019; Xu et al., 2022; Zhou et al., 2022). Here, we have identified *mcr*-1 genes in three polymyxin-resistant *E. coli* isolates. The co-appearance of *mcr*-1 and other resistant genes, especially β-lactamases, is worrying, because of limited therapeutic options.

Several studies have shown an association between the use of polymyxin and the emergence of polymyxin resistance in *K*. *pneumoniae* in hospitals, although the results remain controversial in different countries (Antoniadou et al., 2007; Lee et al., 2009; Zarkotou et al., 2010; Xu et al., 2022; Zhou et al., 2022) In our study, 17 patients received polymyxin therapy during their hospitalizations, 15.6% received topical treatment that involved the use of polymyxin therapy for 7–22 days, 31.2% received PMB IV (25–150 mg) treatment for 2–30 days according to the International Consensus Guidelines for the Optimal Use of polymyxins (Tsuji et al., 2019), and 9 patients were given polymyxin therapy for more than 15 days. Polymyxin-resistant isolates were obtained from 16 of these patients after polymyxin treatment for 2 to 50 days, and only one patient was isolated with the polymyxin-resistant *K. pneumoniae* strain before polymyxin therapy was administered. We also found a divergent prevalence in patient isolates with polymyxin- resistant strains after multiple-time or once- only analysis. In patients’ isolates with multi polymyxin- resistant strains, 15 out of 22 (68.18%) patients were given polymyxin treatment. In reverse, in patients who had polymyxin- resistant strains isolated only once, only 2 of 10 patients (20%) were given polymyxin treatment. From these findings, we were able to demonstrate an association between the use of polymyxin and the emergence of polymyxin-resistant *Enterobacterales*. There is a great risk that long-time dosing might lead to polymyxin resistance among various *Enterobacterales* isolates. Monotherapy with polymyxins should be avoided, with better ways to use them in combination with drugs with synergistic effects or an i.v. spray. According to the host factors of all the collected patients, we concluded that the patients were mainly adults, with multiple complicated comorbid conditions to highly infection-prone environments, and also underwent longtime hospitalization. Fifteen patients who were not given polymyxin therapy also had isolates containing polymyxin-resistant *Enterobacterales*. We may attribute this to their long-term exposure to the environment during hospitalization. Since this was a retrospective study, we did not have the corresponding data about the nosocomial environments. Further studies are therefore needed to demonstrate the prevalence of polymyxin-resistant *Enterobacterales* in hospitals.

## Conclusions

A low prevalence of polymyxin-resistant *Enterobacterales* was found soon after polymyxin therapy was introduced into a Chinese tertiary teaching hospital. The polymyxin-resistant *Enterobacterales* pose a real threat to public health. It is very important for clinical laboratories to detect polymyxin- resistant genes and to characterize the epidemiological trends of high-risk polymyxin-resistant *Enterobacterales* in order to optimize the use of the last-line class of antibiotics. Furthermore, effective infection control measures are urgently needed to prevent further transmission of polymyxin resistance.

## Data availability statement

The datasets presented in this study can be found in online repositories. The names of the repository/repositories and accession number(s) can be found in the article/**Supplementary Material.**

## Ethics statement

The study was conducted according to the guidelines of the Declaration of Helsinki and approved by the Ethics Committee of Shanghai Jiao Tong University School of Medicine (protocol code RJ2022055 and 1 March 2022 approval).

## Author contributions

Project planning, writing of the original draft, CX, XL; genomics analysis, software, and investigation, LZH, XL, HX; project administration; review and editing, LJH, ZY. All authors have read and agreed to the version of the manuscript submitted for publication.

## Supplementary material

Supplementary Figure 1 Phylogenetic tree showing the genomic relationship of the polymyxin-resistant *K. pneumoniae* isolates and public isolates. The phylogenetic tree was constructed based on core genome SNPs and the isolates sequenced in this study was marked with red color in the circle. Altogether, 378 high quality genomes were collected from GenBank database and 28 K*. pneumoniae* polymyxin-resistant genomes with GCA_008728695 *K. pneumoniae* genome as a reference, resulting in 3 distinct clades. The MLST typing results and country information of the isolates were shown in the outer circles from outside to inside.

Supplementary Figure 2 Phylogenetic tree showing the genomic relationship of the polymyxin-resistant *E. coli* isolates and public isolates. The phylogenetic tree was constructed based on core genome SNPs and the isolates sequenced in this study are marked with black triangles. Altogether, 400 high quality genomes were collected from GenBank database and 4 polymyxin-resistant *E. coli* and genomes with *E. coli* GCA_003018455.1_ASM301845v1 genome as a reference. The MLST typing results and country information of the isolates are shown on the right.
