# Supplementary figures and images for: Prevalence and molecular characteristics of polymyxin-resistant Enterobacterales in a Chinese tertiary teaching hospital
Source: Front Cell Infect Microbiol. 2023 Apr 18;13:1118122. doi: 10.3389/fcimb.2023.1118122 (PMC10151768; doi:10.3389/fcimb.2023.1118122)

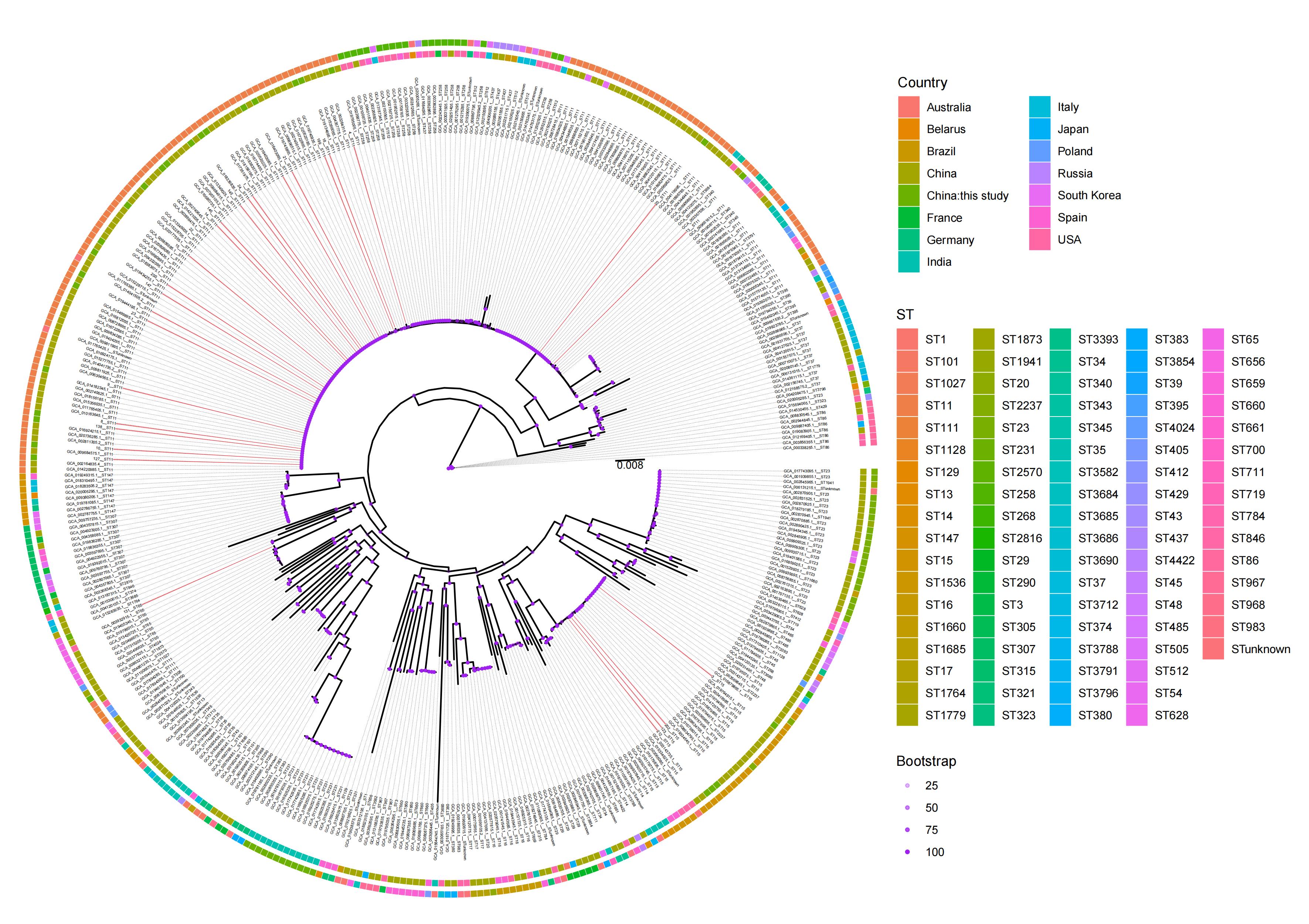

Supplement: Supplementary Figure 1 — Phylogenetic tree showing the genomic relationship of the polymyxin-resistant K. pneumoniae isolates and public isolates. The phylogenetic tree was constructed based on core genome SNPs and the isolates sequenced in this study was marked with red color in the circle. Altogether, 378 high quality genomes were collected from GenBank database and 28 K. pneumoniae polymyxin-resistant genomes with GCA_008728695 K. pneumoniae genome as a reference, resulting in 3 distinct clades. The MLST typing results and country information of the isolates were shown in the outer circles from outside to inside. [file Image_1.jpeg]

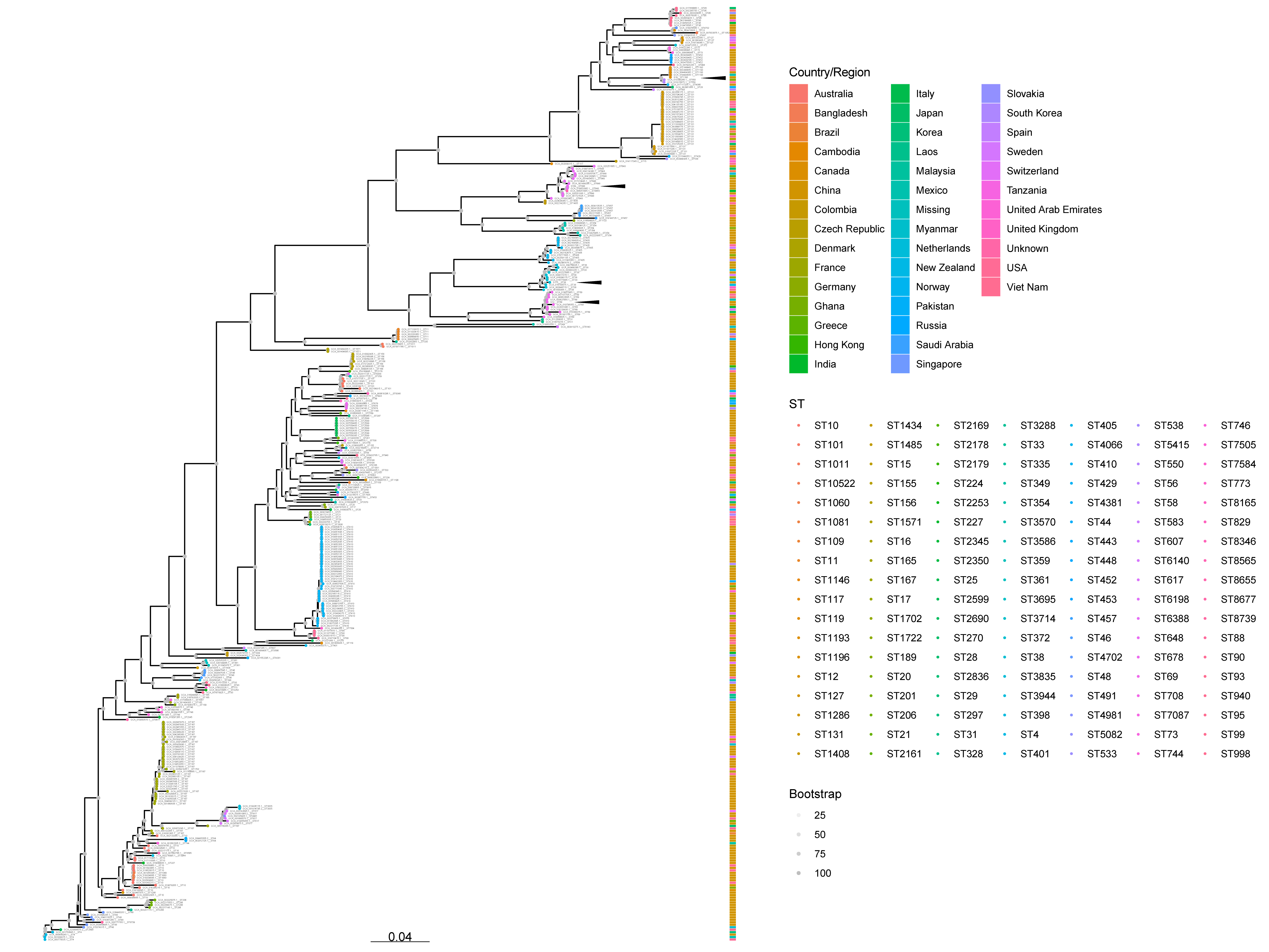

Supplement: Supplementary Figure 2 — Phylogenetic tree showing the genomic relationship of the polymyxin-resistant E. coli isolates and public isolates. The phylogenetic tree was constructed based on core genome SNPs and the isolates sequenced in this study are marked with black triangles. Altogether, 400 high quality genomes were collected from GenBank database and 4 polymyxin-resistant E. coli and genomes with E. coli GCA_003018455.1_ASM301845v1 genome as a reference. The MLST typing results and country information of the isolates are shown on the right. [file Image_2.tif]
